# What do adults want in parks? A qualitative study using walk-along interviews

**DOI:** 10.1186/s12889-022-13064-5

**Published:** 2022-04-14

**Authors:** Jenny Veitch, Nicole Biggs, Benedicte Deforche, Anna Timperio

**Affiliations:** 1 School of Exercise and Nutrition Sciences, Deakin University, Geelong, Australia, Institute for Physical Activity and Nutrition (IPAN), 221 Burwood Highway, Burwood, VIC 3125 Australia; 2 Department of Public Health and Primary Care, Faculty of Medicine and Health Sciences, Ghent University, C. Heymanslaan 10, 9000 Ghent, Belgium; 3 Movement and Nutrition for Health and Performance Research Group, Department of Movement and Sport Sciences, Faculty of Physical Education and Physical Therapy, Vrije Universiteit Brussel, Pleinlaan 2, 1050 Brussels, Belgium

**Keywords:** Design, Adults, Park features, Physical activity, Social interaction

## Abstract

**Background:**

Parks provide opportunities for physical activity and social interaction and are critical for enhancing public health. It is therefore important to better understand the needs and preferences of park features among adults to help park designers to create parks that optimise use. This qualitative study provided an in-depth examination of factors and characteristics that influence visitation, park-based physical activity, and social interaction among adults (19–64 years). We also explored perceptions of parks and park use and impacts of COVID on park usage and needs.

**Methods:**

Participants (*n* = 27, 40.4 years [+ 11.9], 70% female) were interviewed from 2017 to 2020 while walking through one of eight diverse parks located in varying socioeconomic areas of metropolitan Melbourne, Australia. Participants were prompted to discuss their experiences, opinions and preferences regarding park features. All interviews were transcribed verbatim and analysed using NVivo 12 software.

**Results:**

Park features and characteristics highly valued for visitation related to aesthetics and atmosphere, including trees, gardens, spaciousness, and water features. Features most valued for physical activity included walking and bike tracks, basketball rings, nice aesthetics, and sports walls. Features most valued for social interaction included seating and tables, and picnic/barbecue areas.

**Conclusions:**

This study highlights features and characteristics that may be important to prioritise, to encourage active and social park visits among adults. This evidence will help policy and decision makers, urban planners, landscape architects, and local, state, and national government organisations to create parks that support adults to lead healthy and active lives. Future research should examine the relative importance of the features identified in this study to inform future park design/redesign.

**Supplementary Information:**

The online version contains supplementary material available at 10.1186/s12889-022-13064-5.

## Introduction

Creating cities with high-quality parks is a global priority for enhancing the health of current and future generations [1, 2]. Parks can facilitate a wide range of free physical activities and support social interaction and social contact [3, 4]. The availability of high-quality parks that support physical activity is critical as physical inactivity is responsible for more than five million deaths per year globally. Currently in Australia, approximately 55% of adults do not do enough physical activity to meet Australian Government recommendations [5, 6]. As well as physical activity, visiting parks has been shown to facilitate psychological and social health benefits such as decreased stress, anxiety, and depression [7–9], and it has been suggested that to enhance the well-being of park visitors it is necessary to provide park features that encourage social interaction [10]. Social interaction is essential for positive mental health.

Despite the many benefits of park use, studies have shown in Australia [11] and elsewhere [12] that parks are generally under-utilised. In addition, most park users have been observed in sedentary activities such as sitting or engaging in low levels of physical activity [11–15]. For example, a national study of US parks reported that 62% of visitors observed in parks were being sedentary [16]. Review studies [12, 13], and a previous study of park visitors in Melbourne, Australia [11] have also shown youth to be more active in parks than adults. It is therefore critical to identify ways to facilitate increased park visits and support adult park visitors to be active and social during their park visits.

Previous research has shown certain features to be associated with park-based physical activity among adults [17, 18]. For example, in a study of adults in Denmark, park size, walking/cycling paths, wooded areas, water features, lights along trails, pleasant views, bike racks and parking lots were positively associated with self-reported physical activity in the closest urban green space [19]. Condition and cleanliness of park features have also been found to be associated with higher levels of observed park-based physical activity [15]. Natural experiment studies have shown that improving or adding new park features can result in increased park visits and park-based physical activity among adults [20–22], and are a cost-effective way to increase park-based physical activity [23]. Self-reported park satisfaction has also been shown to be positively associated with park visits among adults [24], therefore understanding what park features are most important for adults and including these features in future park refurbishments is likely to increase park satisfaction and lead to increased park visits. To our knowledge, studies examining how parks may be designed to encourage social interaction are scarce and this is an important research gap as previous research has highlighted that park features that support social interaction may differ to features required for other park behaviours [25].

Limited qualitative studies have provided an in-depth exploration of park features that are important for not only encouraging park visitation among adults but also encourage physical activity and social interaction during park visits. A small number of qualitative studies have examined this topic with youth and older adults [26–29], however, different age groups have different needs [18, 30, 31] and parks often lack features and amenities that meet the needs of potential users across all age groups. It is therefore important to examine this topic with adults.

The aim of this qualitative study was to explore adults’ (19–64 years) perceptions of park features and characteristics that influence their park visitation, park-based physical activity, and social interaction using walk-along interviews**.** We also explored perceptions of parks and park use and impacts of COVID on park usage and needs. Walk-along interviews involve conducting interviews while walking through the park, which creates an opportunity to examine the topic in-situ. This contrasts with other qualitative methodologies such as individual and focus groups interviews which require participants to recall past experiences. Walk-along interviews have been used to gain in-depth insights on park features among children [26], adolescents [28, 29], and older adults [27], but have not been used to explore park features among adults. This research will provide an in-depth understanding of important park features and characteristics for adults and will help stakeholders such as park planners and designers to create parks that encourage adults to visit parks and be active and social during their visit.

## Methods

Walk-along interviews (*n* = 27) were completed in eight parks across metropolitan Melbourne, Australia between September 2017 and July 2020. Nine interviews were conducted during the COVID-19 pandemic, but not during lock-down conditions. The parks were located 3–57 kms from Melbourne’s central business district in varying socio-economic status areas (SES; 2 parks in low SES areas, 3 in mid SES, 3 in high SES) as defined by the Australian Bureau of Statistics Socioeconomic Index for Areas [32]. Parks were selected to represent a variety of sizes (range 1–30 ha), conditions, and amenities to ensure that potentially positive and negative park characteristics could be observed during the interview. For example, some parks had few amenities (e.g., no playground or toilets) and/or lacked maintenance (worn equipment, overgrown grass), whilst others had a high standard of maintenance with a wide range of features and amenities. See Supplementary Table 1 for a description of the park amenities.

Recruitment was conducted in two waves - wave one was via ProjectPARK (parents), and wave 2 occurred 26 months later and recruited general adults. Participants were recruited through multiple methods including in park recruitment (*n* = 18), flyers posted around the University (*n* = 2) and a doctor’s clinic (*n* = 1), word of mouth (*n* = 3), and Facebook (*n* = 3). For in park recruitment, research staff approached park visitors who appeared to be aged between 19 and 64 years, explained the study, and invited them to participate. Adults were required to speak English and provide written consent prior to the interview. Eleven adults who were approached declined to participate. For all other methods of recruitment, participants contacted the study team, who explained the study. If they wished to participate, a day and time was arranged to meet the participants at one of the eight parks in the study (usually the one located closest to their home). Twelve of the 27 participants were recruited via ProjectPARK, with 11 completing their interview after their child, and one completing the interview at the same time (but separate) [26, 28]. The same interview schedule described below was used for these interviews. Recruitment ceased when saturation was reached. Eight research assistants completed the interviews, with one researcher completing 52% of interviews (*n* = 14).

Firstly, to describe our sample participants and their usual park use behaviours they completed a brief self-report survey on demographics and their usual park visitation behaviours over the past 3 months (Table 1). They were asked to report their age, sex, dog ownership, country of birth, highest level of education, parental status (number of child(ren)/grandchild(ren)), physical activity levels, frequency and duration of park visits, activities undertaken and physical activity levels whilst at the park, accompaniment and mode of transport to the park, and frequency of engaging with people they either did or did not know when at the park. Secondly, participants completed a semi-structured walk-along interview whilst walking through the park with the researcher. Sometimes the researcher suggested where to walk and at other times the participant directed where they wanted to walk. Most areas of the park were viewed or walked through. Participants who had visited the park previously were firstly asked to describe why they visited the park, whereas participants who had never visited the park were asked to walk and look around the park to familiarise themselves with the park and then talk about things they may like to do in the park. Participants were then prompted to discuss their experiences and opinions through the following questions: How does being in a park make you feel?; What do you like/dislike about this park?; What makes you want to visit here and be active and social here?; How would you suggest changing the park to make it better or make you want to visit, be active or social?; Please describe your ideal or perfect park?. The nine participants who completed their interview during the COVID-19 pandemic were also asked if their park usage or park needs had changed due to the pandemic. Both the researcher and the participant carried a voice recorder throughout the interview and interviews ranged between 7 and 28 min (mean 12.6, + 5.2 mins). The study was approved by the Deakin University Human Ethics Advisory Group (HEAG-H 94_2017) and all methods were performed in accordance with the relevant guidelines and regulations.

**Table 1:** Demographic and park visitation characteristics of participants

	***N*** = 27
Had previously visited park where interview conducted, n (%)	16 (59.3%)
Age, mean [SD]	40.37 [11.9]
Sex, Female, n (%)	19 (70.4%)
Country of birth, n (%)	
Australia	16 (59.3%)
Other	10 (37.0%)
Missing	1 (3.7%)
Dog owner, n (%)	9 (33.3%)
Highest level of education, n (%)	
Low (did not complete high school)	1 (3.7%)
Medium (year 12/trade/certificate)	6 (22.2%)
High (university or tertiary qualification)	20 (74.1%)
Child(ren), < 12 months, n (%)	2 (7.4%)
Grandchild(ren), < 12 months, n (%)	1 (3.7%)
Child(ren), 1–18 years, n (%)	15 (55.6%)
Grandchild(ren), 1–18 years, n (%)	2 (7.4%)
Years living at current address, mean [SD]	8.7 [8.5]
Usual frequency of park visits in past 3 months, n (%)	
About once per month	1 (3.7%)
2–3 times per month	5 (18.5%)
About once per week	6 (22.2%)
2–3 times per week	11 (40.7%)
Daily	4 (14.8%)
Usual duration of park visits in past 3 months, n (%)	
< 30 min	4 (14.8%)
30–59 min	12 (44.4%)
1h hours	10 (37.0%)
Missing	1 (3.7%)
Usual activity levels when at the park in past 3 months, n (%)	
Mostly sitting/light activities	17 (63.0%)
Mostly moderate activities	3 (11.1%)
Mostly vigorous activities	2 (7.4%)
Missing	5 (18.5%)
Usual accompaniment when at the park in past 3 months, n (%)^a^	
Alone	12 (44.4%)
Other adult family members	6 (22.2%)
Grandchild(ren)	1 (3.7%)
Children	15 (55.6%)
Friend(s)	10 (37.0%)
Dog	7 (25.9%)
Other (partner)	3 (11.1%)
Usual mode of transport to park in past 3 months, n (%)^a^	
Walked	18 (66.7%)
Jogged	3 (11.1%)
Cycled	2 (7.4%)
Public Transport	3 (11.1%)
Car	9 (33.3%)
Frequency of talking to people in park in past 3 months never met previously, n (%)	
Never/rarely (0–1 times)	10 (37.0%)
Sometimes (2–5 times)	14 (51.9%)
Most of the time/always (6+ times)	3 (11.1%)
Frequency of talking to people in park in past 3 months that already knew, n (%)	
Never/rarely (0–1 times)	11 (40.7%)
Sometimes (2–5 times)	11 (40.7%)
Most of the time/always (6+ times)	5 (18.5%)
Frequency of participating in a social event in park in past 3 months, n (%)	
Never/rarely (0–1 times)	20 (74.1%)
Sometimes (2–5 times)	7 (25.9%)
Most of the time/always (6+ times)	0

^a^multiple responses possible

### Data analysis

Descriptive statistics from the survey data were calculated using Stata Statistical Software version 15 (Stata Corp., College Station, TX, USA). Audio recordings from the walk-along interviews were transcribed verbatim and entered into NVivo 12 Plus for analysis (QSR International Pty Ltd., Melbourne, Australia). Qualitative data analyses were performed using inductive content analysis guided by a summative approach [33]. A preliminary coding framework was established based on the interview questions described above (e.g., what liked about this park). Possible responses (e.g., trees, location) were in the coding framework; however, the framework was revised and adapted throughout the coding process as new content emerged. This type of analyses has been performed with similar studies involving walk-along interviews with various age groups [26–28]. Interviews were read carefully and coded into sub-categories and groups. All interviews were coded by a single researcher (NB) and 50% were cross coded by a second researcher (JV). Any disagreements were discussed between NB and JV until a conclusion was reached.

## Results

Overall, 27 adults completed interviews across the eight parks (9 interviews in each SES area). Participants were aged 21–61 years (mean = 40.4 (+ 11.9) years), 70% were female, and over half (59%) had previously visited the park where the interview was conducted. Nearly three quarters (74%) were tertiary educated, 56% were parents of children aged 1–18 years and 7% had grandchildren (1–18 years). The most common mode of transport to travel to the park was walking (67%), 78% visited parks at least once per week in the past 3 months, and 63% reported their usual park-based activity to be mostly sitting or light intensity activities (Table 1).

### Activities performed and how they feel when in the park

The five main activities they could envisage doing or main reasons for visiting this park were to go for a walk/run, sit or relax, supervise children, have a picnic or eat lunch, and meet with family or friends. All participants gave positive responses when asked how being in a park made them feel with most saying it made them feel relaxed, less stressed and refreshed. Others said it cleared their mind, made them feel happy, close to nature and energised.

*It centres me. It’s good for my soul. There are so many apartments going up in X now. Often the thought of it makes me feel anxious, but just coming here helps with that anxiety. Again, if we didn’t have these places, they would be building apartments here, and it would be horrendous. So, I think this space personally is very important for me, mentally and emotionally (Female, 57 years, park in high SES area)*

*I feel very relaxed, away from the noise and the pollution, and close to nature.. … I mean I'm not that stressful person, but if there is any kind of pressure or stress you know you can sort of come into park and release (Male, 57 years, park in high SES area)*

### Park features: Likes, dislikes, preferences, and suggestions for change

#### What they liked about the park

When asked to describe what they liked about the park the most common responses related to the theme of aesthetics and atmosphere. This included trees, nature and greenery, gardens, the quiet, peaceful and private atmosphere, the sense of spaciousness, and water features.

*It’s got a very nice aesthetic. All the plants around. There’s quite a few more than your regular park. The gardens, there’s a large variety of plants and it’s very nice to look at. Contributes to the relaxing atmosphere, I’d say (Male, 21 years, park in high SES area)*

Other common responses related to the theme of amenities such as the provision of tables and seating, and a diversity of park features to ensure sufficient amenities for all age groups. Play equipment was also frequently mentioned by parents, as well as the park being of a large size.

*It’s very green, so it’s like very lush, and a lot of space, and there’s many facilities here. For children there are a lot of things around in the park that they can do. There’s also a lake, a small pond. … .. and there’s a nice walking trail also, so if you want to go walking in the evening, it’s a nice park for that (Female, 32 years, park in mid SES area)*

#### What they disliked about the park

Lack of safety was what participants disliked most about the park, although this was only mentioned by females. This included a lack of fencing around the playground, and the presence of ‘undesirable or suspicious people’.

*You know the people who take drugs they throw the syringes and sometimes you know they are around here in the evening time especially. So it’s a bit unsafe for us to come and also with kids it’s especially more unsafe because I feel they look at them and then maybe they are curious to know what they are doing (Female, 43 years, park in low SES area, parent)*

Other frequently mentioned factors included lack of maintenance such as rubbish and unclean or smelly toilets, park being too crowded, lack of toilets, little or no shade, traffic or noise, lack of parking, too much grassy space, and play equipment not designed for a range of ages.

*When I come sometimes of a morning there’d be people who would have a barbecue and I cannot believe it, and often it would be right near the bin, and they would have left everything all over the table. Empty bottles of beer, so it’s not very nice thought to think that there were people here who were drinking lots of alcohol and have left lots of rubbish (Female, 57 years, park in high SES area)*

*Not just this park, but parks in general, I dislike it when it’s near main roads. It gets a bit noisy from cars. I like it when it’s a bit quieter, it’s just easier. If I’m listening to a podcast, reading a book, it’s much easier to focus without the cars making a bit of noise. So I mean, it’s kind of hard to get around with a lot of main roads around this area, but that’s probably the worst part (Male, 22 years, park in mid SES area)*

#### What they would change to encourage visitation

Participants most frequently mentioned that the provision of more shade and shelter would encourage them to visit the park more often.

*Maybe a bit more shelter. I just noticed, there’s one rotunda … . but I think in summer you’d probably want a little bit more structured shelter, built shelter. The trees would probably most likely provide a lot of it, but also in winter, you want to be able to, if you’re going to sit somewhere, you want to have a couple of options (Female, 60 years, park in high SES area)*

Improved safety was the next most common response, followed by improved maintenance/cleanliness, more gardens, flowers and trees, a café or coffee cart, and more toilets. Other less common suggestions included the provision of a herb garden, oversize chess sets, ornamental elements and statues, bouldering or climbing structures, in-ground trampolines and water play elements. Parents were particularly concerned about improving safety, and they suggested adding fencing around the playground or park

*The only main thing would be the fencing. As my kids get older it's not so much of an issue for me but I know friends with younger kids tend to not come here because of that fact. They can't enclose them and they've got like a couple of kids that are runners. So, you need to kind of trap them somewhere (Female, 42 years, park in low SES area, parent)*

Increased shade and improved maintenance were also frequently mentioned by parents, whereas gardens/flowers and shelter were mentioned more frequently by non-parents.

#### Features that encourage physical activity

A walking path/bike track was the feature most frequently mentioned as encouraging active use of the park. Participants liked paths that were multi-directional and wide enough to allow space for people walking in both directions.

*So, I like how it [walking path] is not flat lined, but it has gentle slopes up and down, so you can increase your pace of walking and running, and then you can slow down if you want to (Male, 38 years, park in mid SES area)*

A basketball ring/court, nice aesthetics, sports wall, and playground were also highlighted as important features. Less frequently mentioned but potentially important aspects included having points of interest to look at within the park and things to look at along the paths, presence of others exercising, sloping land, variety of equipment and amenities, and facilities for dogs to be allowed off lead.

*I’m more driven to do physical activity looking at other people running and cycling around. So, it’s kind of like motivating me to also do something like that. Having a lot of people around you playing basketball and cycling makes me motivated when I see them doing that (Female, 32 years, park in mid SES area)*

When asked to suggest changes to the park to encourage them to be more active, the most common response was the provision of fitness equipment. This was of particular importance to parents.

*Park fitness gym sort of equipment. Like particularly like for mum's, if your kid's riding round on a bike you could just sit and do a couple of little exercises while they're doing it (Female, 42 years, park in low SES area, parent)*

Other suggestions included the provision of a large grassy open space area, separated walking and bike tracks, and a basketball court. Less frequently mentioned but potentially important inclusions were a bike lock station, large park size, line markings on courts for different sports or activities, organised park runs/walks, a skate ramp, and a separate zone for exercising.

*I like being active in a bigger area. Running around this park, you’d probably get a bit bored, just the small laps over and over again. Open spaces. Having like a nice grass clearing would be good. Enough space to kick the footy with a friend or something like that. Possibly like a basketball ring as well. I reckon that would definitely encourage me [to be active]. (Male, 21 years, park in high SES area)*

#### Features that encourage social interaction

Seating, tables, and picnic and barbecue areas were the most frequently mentioned features for encouraging social interaction in the park. To ensure parents could interact with other parents, it was suggested that spaces for parents to sit and talk whilst supervising their children were provided and it was also necessary that the park accommodated children’s needs and children were entertained. Park aesthetics including the presence of grassy open spaces, and a private, quiet and peaceful atmosphere with trees, nature and greenery were also considered important for encouraging social interaction.

*Interviewer (What makes you want to hang out with other people at this park)*

*Response: It’s a very pleasant area to sit in. It’s got a nice, relaxing vibe. There are a lot of places to sit, so probably won’t be short on a seat. I can see a barbecue over there, which is always nice. It’s good cooking up lunch in the park, it’s always fun. Yeah, and it’s kind of nearby as well (Male, 21 years, park in high SES area)*

*Interviewer (What makes you want to hang out with other people at this park)*

*Response: There’s a lot of facilities, like there’s a lot of people around, you can just bring your mat here and have some time. It’s very spacious, it’s kind of a park where you can just laze around on the grass (Female, 32 years, park in mid SES area)*

When asked to suggest changes to the park to encourage more social interaction the most frequent responses were the provision of shade and shelter, barbecues, a café, food van or shop, and seating and tables.

*Interviewer (So you'd like a little coffee shop a bit closer?)*

*Respondent: Yeah and it means like a group of parents they love to come over here, order the hot coffee, sit together, watch their children play (Female, 44 years, park in high SES area, parent)*

*Maybe probably just to do with the seating areas as well. Maybe bigger ones, or more under cover ones. Possibly more barbecues is a good idea. It depends a lot on location as well. If all my friends lived around here, this would be a pretty great place to go (Male, 21 years, park in high SES area)*

Public toilets, secluded areas, more trees, and areas sectioned off for children were also mentioned. For non-parents, a BBQ and shelter were the two most popular answers, whereas for parents, shade and a coffee cart were mentioned more frequently.

### Description of their “Perfect Park”.

**Fig. 1:**
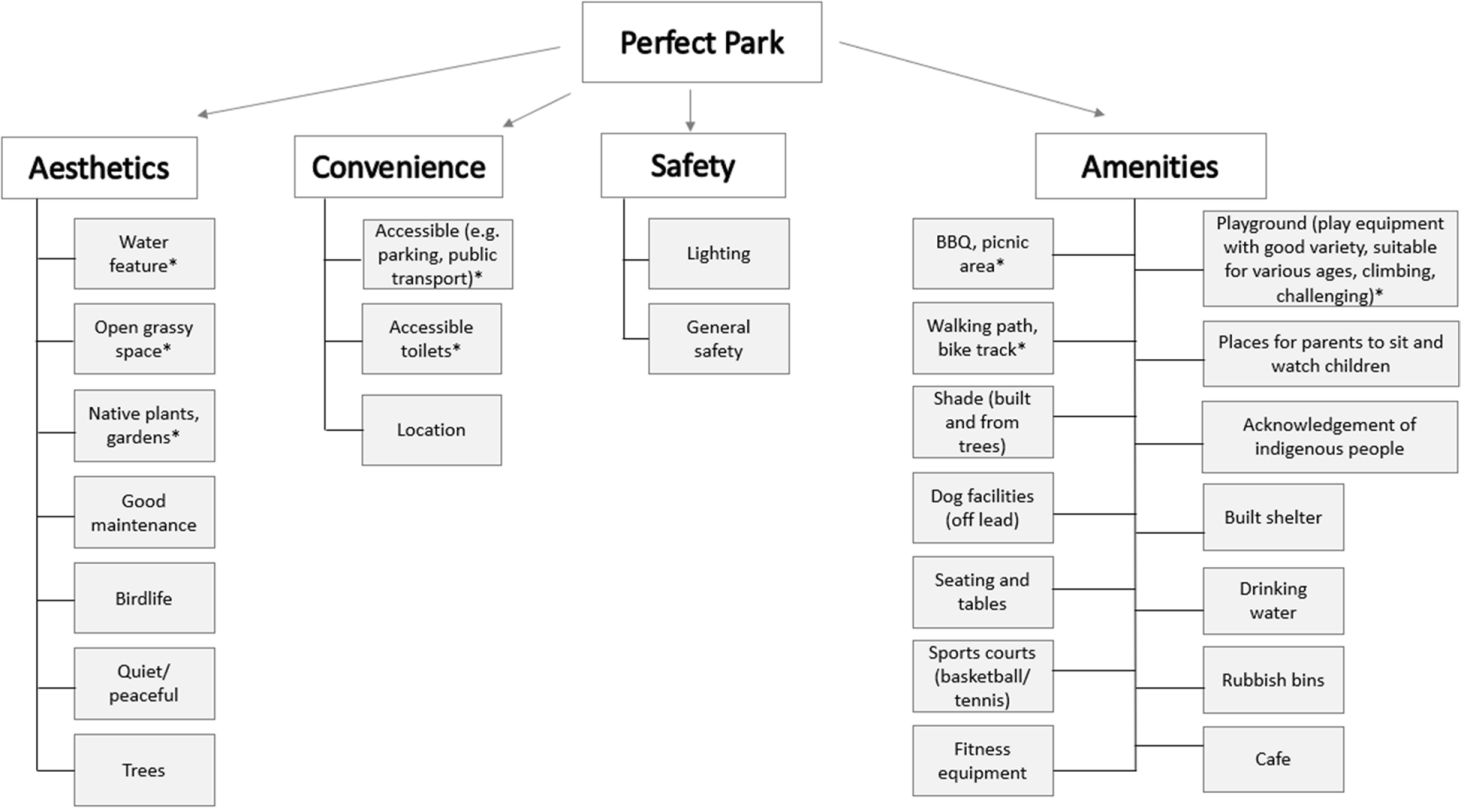
Perfect park features. *Indicates that it was mentioned by at least 5 participants

When asked to describe their “perfect park”, four main sub-themes emerged; convenience, amenities, aesthetics, and safety (Fig. 1). The most frequently mentioned features included it being convenient and accessible with the provision of car parking and public transport, having suitable amenities such as toilets, a barbecue/picnic area, play equipment (good variety, suitable for various ages, climbing, challenging), a walking/bike path, having nice aesthetics such as a water feature, open grassy space, and native plants and gardens; and being safe (Fig. 1).

*I enjoy seeing people walk their dogs. I just enjoy watching or interacting with the dogs. So, I guess a spot where people can easily access for dog walks. The size as well, there needs to be enough area, away from noise, traffic, and cars. Water features, ponds, fountains, spots to sit, and accessibility. It’s a bit frustrating when all the benches are filled up, and you can’t sit down somewhere. And a bit of nature, I always enjoy nature it is probably my biggest thing (Male, 22 years, park in mid SES area)*

*For me, the perfect park would be one which has a walking trail and a cycle trail, both. And there is more of trees and flowers in the park, and more greenery, and there’s more places for us to sit, and also facilities to do physical activity like for the kids to play around. And a basketball court or any kind of a sport facility (Female, 32 years, park in mid SES area)*

*It'd probably have a playground for kids, with a flying fox or something, 'cause I think that'd be fun for adults as well. It'd have, it's just I guess I'm biased because I don't really use netball courts or oval, softball or cricket, and stuff like that, but for other people those would be. I think lots of trees are great. I love trails, so I think just anything that keeps as much nature as there can be in the park, so whether that's like paths that aren't flattened out completely, or the gravel, so it just seems more natural as opposed to concrete. Lots of drink taps throughout the park. And bins, because there's a lot of parks nowadays that they don't have many bins, or they might not even have any, and that's a pain if you're walking your dog and you need to dispose of something. And off-lead park for dogs. But yeah, I love just really manicured ovals, well maintained, and just lots of trees and greenery. Barbecues and facilities, and just like tables, and a shelter would be great as well (Female, 24 years, park in high SES area)*

For parents, the most important features were accessibility (i.e., car parking/public transport) and the provision of accessible toilets, closely followed by play equipment and shade. Whereas for non-parents, walking paths, water features, and green space, nature and gardens were most frequently mentioned.

*The botanical gardens, I love those. Lots of interesting nooks that you could, be like hidden away that you can find if you’re walking around on a path. Hidden water features, things that sort of surprise you as you’re coming around a path. Even if you know the park well, you still find it really lovely to walk around a corner and find a bit of a garden that’s just not your normal garden, it’s got some feature (Female, 60 years, park in high SES area)*

### Impact of COVID-19 on park usage and park needs

Most of the nine participants who were interviewed during the pandemic reported that they had used parks more regularly since the pandemic began. They spoke about how parks were particularly important as they were spending more time at home so appreciated being able to leave the house and go for a walk. Parks were also a place where they could safely socially distance and provided an opportunity to have fresh air, be in nature, have a change of environment, and relax.

However, one participant said their park usage had decreased and one said their frequency of visitation was unchanged.

*I’ve visited here [parks] more often, just wanting to have a bit of head space (Female, 57 years, park in high SES area)*

*I’ve spent more time going to parks by myself. It’s a pretty big thing, and in a sense with Covid, being socially distancing, need the room to be able to have, if there’s a fair few people there, then you can still socially distance, and interact at a distance at the same time (Male, 22 years, park in mid SES area)*

*Yes, we are definitely considering the Covid thing, and we are being indoors most times. We’re now driven to go out and spend some time outdoors being in the park, and hanging out in the park so we get some more fresh air and all those things. So we’re more inclined to go out these days (Female, 32 years, park in mid SES area)*

*I just like being out in nature more because I’m at home more. So I think I just take more pockets of time, quietly here, whereas before it was go to work, walk the dog, do this. Whereas now it’s like, come, just chill out for a bit (Female, 32 years, park in mid SES area)*

## Discussion

This qualitative study explored park needs and preferences regarding park features and characteristics that may encourage park visitation, park-based physical activity, and social interaction among adults (19–64 years). It was unique in its exploration of features that influence three specific behaviour outcomes, park visitation, park-based physical and social interaction. Participants discussed multiple features that they liked and disliked and different features emerged as being important for the three outcomes highlighting the importance of considering multiple behaviours and uses of the space when designing parks. This is consistent with previous research that highlighted features that support social interaction may differ to features required for other park behaviours [25].

Overall, aesthetics and atmosphere were what participants liked most about the parks, particularly a quiet, peaceful, and private atmosphere, with a sense of spaciousness and the provision of trees, nature, greenery, gardens, and water features. This is similar to preferences by older adults [27, 34] which reinforces the need to prioritise the greening of parks and public spaces to include trees and gardens and a feeling of being in nature rather than simply an open space with amenities. This is a critical consideration for park design, as well as for on-going maintenance to ensure plants and gardens thrive and are well cared for [28]. Park aesthetics, including the presence of grassy open spaces, and a private, quiet, and peaceful atmosphere with trees, nature and greenery were also considered important for encouraging social interaction. This is consistent with previous research among older adults [34], and large grassy open space has also been shown to be an important feature for promoting social interaction among adolescents [35]. In addition to the creation of this natural environment, participants expressed the need for a diversity of park features to meet the needs of all age groups and amenities such as shade and shelter, barbecues, a café, and seating and tables to increase social interaction. These amenities were also discussed as being important for promoting social interaction during walk-along interviews with older adults [27] and adolescents [28]. For parents, the provision of play equipment as well as the park being of a large size were important as it helped to ensure that the park accommodated children’s needs and also supported social interaction as it enabled parents to interact with others while their children were occupied.

Lack of safety was what they were most concerned about, although this was only mentioned by females who comprised 70% of our sample. Safety concerns were varied and included a lack of fencing around the playground, and personal safety, with many expressing concerns about the presence of undesirable or suspicious people. Although, previous research in the US found perceptions of park safety were not associated with the number of people observed in the park [36], it is important for future studies to explore ways to increase perceptions of park safety. Installing fencing around playgrounds may be a feasible change that can improve perceptions of safety for parents of young children; however, safety from other people is a more complex issue that may need to be addressed via multiple angles. Lack of maintenance was also frequently mentioned as discouraging visitation and improved maintenance was suggested to increase visitation. A review of qualitative studies found that poor maintenance and condition may discourage park use and negatively affect perceptions of safety and park quality [37]. A case study in Canada reported greater cleanliness and condition were generally associated with higher observed physical activity levels within parks; however, results were not reported via age group [15]. Furthermore, previous research in Melbourne found good maintenance and feelings of safety were the two most important features for engaging in park-based physical activity among adults < 60 years [30]. Interestingly, in the current study good maintenance was not raised in relation to characteristics that would support physical activity although nice aesthetics was mentioned as being important.

A walking path/bike track was the feature most frequently mentioned as encouraging active use of the park. Participants liked paths that were multi-directional and wide enough to allow space for people walking in both directions. Previous studies have observed park users overall to be most active on paths [15], and it is acknowledged that walking paths are critical park features for older adults [27, 38]. Previous research has also shown walking/jogging paths to be more important for park-based physical activity among adults currently meeting physical activity guidelines compared with those not meeting guidelines [30]. It is therefore important for park designers to prioritise the inclusion of paths; however, the micro-scale elements such as width, direction and aesthetics are essential to make them appealing and potentially encourage use among those who are not regularly active so this needs to be a focus of future research. A basketball ring/court, nice aesthetics, sports wall, and playground were also highlighted as important in the current study. These have also previously been highlighted as important for promoting park-based physical activity among adolescents [35]. Previous research has highlighted that although playgrounds are the locations where children are most likely to engage in higher levels of physical activity [39], existing playgrounds are not designed to encourage adults to be active. Future studies should explore playground elements that may encourage adults who visit playgrounds with their children to be more active. When asked to suggest changes to the park to encourage them to be more active, the most common response was the provision of fitness equipment. This was of particular importance to parents. Previous studies examining the impact of installing fitness equipment have shown mixed results [21, 40–42], so future studies are needed to understand the required design elements of fitness equipment to optimise use across all demographic groups.

### Strengths and limitations

The main strength of this study was the walk-along methodology that created an interplay between the environment (both social and physical), the researcher, and the participant which led to in-depth information and new insights. The broad age range (21–61 years), the inclusion of both parents and non-parents, and the inclusion of parks of varying size and amenity were further strengths that allowed for the exploration of a diverse range of ideas and experiences. However, it is possible that the variation in features present in the parks where the interviews were conducted influenced the frequency some features were discussed. In addition, one researcher coded all interviews with 50% being cross coded, which ensured consistency and accuracy across the analysis. However, there are several limitations to acknowledge. Although there was an even split of interviews performed in parks located in low, mid, and high SES areas, 74% of participants had a high level of education. Nine interviews were completed after 1 month of the initial COVID-19 lockdown period in Melbourne, which may have influenced participants’ park visitation habits and views. Further, most participants were female, recruited from within the park, and were regular park visitors, with 78% visiting parks at least once per week in the past 3 months. Therefore, future studies should explore the views of males and non-frequent park visitors. Finally, this study focused on features within the park, so future studies may wish to examine factors within the neighbourhood surrounding the parks that are important for encouraging park use such as reducing noise from traffic, and factors supporting park access such as road crossings, footpaths, and public transport.

## Conclusion

The results of this study suggest that priority should be given to greening of parks to create a feeling of being in nature and a sense of peace, quietness, and spaciousness to make them more appealing to visit, along with providing walking/bike tracks, basketball rings, sports walls, and playgrounds to support physical activity and seating, tables, cafés and picnic and barbecue areas to facilitate social interaction. Ensuring parks are harnessed and meet multiple needs of users is critical. Future research should examine the relative importance of the features identified in this study to inform future park design/redesign.

## Supplementary Information

**Additional file 1.**

## Data Availability

The datasets used and/or analysed during the current study are available from the corresponding author on reasonable request.
