# Supplementary material for: What do adults want in parks? A qualitative study using walk-along interviews
Source: BMC Public Health. 2022 Apr 14;22:753. doi: 10.1186/s12889-022-13064-5 (PMC9008398; doi:10.1186/s12889-022-13064-5)
Supplement: Supplementary file 1 — Additional file 1. [file 12889_2022_13064_MOESM1_ESM.docx]

|  | **SES** | Size | Adven  ture play | Play  ground | BMX/  skate ramps | Ropes course | Climbing structure | Path | Land-scaping | Pond | Shady  trees | Built shade | Water Play | BBQ | Picnic tables | Toilets | Ovals | Cricket Nets | BBall court | Fitness Equip | Tennis court | Table  tennis |
| --- | --- | --- | --- | --- | --- | --- | --- | --- | --- | --- | --- | --- | --- | --- | --- | --- | --- | --- | --- | --- | --- | --- |
| 1. | Low  (35km from CBD) | 3.7ha | No | Yes - 2 | Yes | No | Yes | Yes | No | No | Yes | Yes | No | Yes | Yes | Yes | Yes | No | Yes – half court | No | No | No |
| 2. | Low  (57km from CBD) | 29.7ha | Yes | Yes - 2 | Yes | Yes | No | Yes | Yes | No | Yes | Yes | No | Yes | Yes | Yes | Yes | Yes | Yes | No | No | No |
| 3. | Mid  (19km from CBD) | 6.5ha | Yes | Yes - 2 | Yes | No | Yes | Yes | Yes | Yes | Yes | Yes | No | Yes | Yes | Yes | No | No | Yes | No | No | Yes |
| 4. | Mid  (13km from CBD) | 1.7ha | Yes | Yes | No | No | Yes | Yes | Yes | No | Limited | Yes | Yes | Yes | Yes | Yes | No | No | Yes – half court | No | No | No |
| 5. | Mid  (4km from CBD) | 1.0ha | No | Yes | No | No | No | Yes | Yes | No | Yes | No | No | Yes | Yes | No | No | No | Yes – half court | No | No | No |
| 6. | High  (8km from CBD) | 1.2ha | No | No | No | No | No | Yes | Yes | Yes | Yes | Yes | No | Yes | Yes | No | No | No | No | No | No | No |
| 7. | High  (14km from CBD) | 16.6ha | No | Yes | No | No | Yes | Yes | No | No | Yes | Yes | No | Yes | Yes | Yes | Yes | Yes | No | No | No | No |
| 8. | High  (3km from CBD) | 1.9ha | Yes | Yes | No | Yes | Yes | Yes | Yes | No | Yes | No | Yes | Yes | Yes | Yes | No | No | No | No | No | No |

**Table S1: Characteristics of parks where interviews were conducted**
